# The Influence of Mechanical Alloying and Plastic Consolidation on the Resistance to Arc Erosion of the Ag–Re Composite Contact Material

**DOI:** 10.3390/ma14123297

**Published:** 2021-06-15

**Authors:** Dariusz Kołacz, Stanisław Księżarek, Piotr Borkowski, Joanna Karwan-Baczewska, Marcin Lis, Małgorzata Kamińska, Barbara Juszczyk, Joanna Kulasa, Aleksander Kowalski, Łukasz Wierzbicki, Krzysztof Marszowski, Mariusz Jabłoński

**Affiliations:** 1 Łukasiewicz Research Network-Institute of Non-Ferrous Metals, 5 Sowińskiego Str., 44-100 Gliwice, Poland; stanislawk@imn.gliwice.pl (S.K.); marcin.lis@imn.gliwice.pl (M.L.); malgorzata.kaminska@imn.gliwice.pl (M.K.); barbara.juszczyk@imn.gliwice.pl (B.J.); joanna.kulasa@imn.gliwice.pl (J.K.); aleksander.kowalski@imn.gliwice.pl (A.K.); lukasz.wierzbicki@imn.gliwice.pl (Ł.W.); krzysztof.marszowski@imn.gliwice.pl (K.M.); 2 Department of Electrical Apparatus, Lodz University of Technology, 18/22 B. Stefanowskiego Str., 90-924 Łódź, Poland; piotr.borkowski@p.lodz.pl (P.B.); mariusz.jablonski@p.lodz.pl (M.J.); 3 Faculty of Non-Ferrous Metals, AGH University of Science and Technology, Al. A. Mickiewicza 30, 30-059 Krakow, Poland; jokaba@agh.edu.pl

**Keywords:** contact material, composite, mechanical alloying, pressing, sintering, extrusion, arc erosion, electrical properties

## Abstract

The article presents the influence of mechanical alloying and plastic consolidation on the resistance to arc erosion of the composite Ag–Re material against the selected contact materials. The following composites were selected for the tests: Ag90Re10, Ag95Re5, Ag99Re1 (bulk chemical composition). Ag–Re materials were made using two methods. In the first, the materials were obtained by mixing powders, pressing, sintering, extrusion, drawing, and die forging, whereas, in the second, the process of mechanical alloying was additionally used. The widely available Ag(SnO_2_)10 and AgNi10 contact materials were used as reference materials. The reference AgNi10 material was made by powder metallurgy in the process of mixing, pressing, sintering, extrusion, drawing, and die forging, while the Ag(SnO_2_)10 composite was obtained by spraying AgSniBi alloy with water, and then the powder was pressed, oxidized internally, sintered, extruded into wire, and drawn and die forged. The tests of electric arc resistance were carried out for loads with direct current (DC) and alternating current (AC). For alternating current (I = 60 A, U = 230 V), 15,000 switching cycles were made, while, for constant current 50,000 (I = 10 A, U = 550 V). A positive effect of the mechanical alloying process and the addition of a small amount of rhenium (1% by mass) on the spark erosion properties of the Ag–Re contact material was found. When DC current of 10 A was used, AgRe1 composite was found to be more resistant than commonly used contact materials (AgNi10 and Ag(SnO_2_)10).

## 1. Introduction

The development of electrical engineering and electronics stimulates changes in design and manufacturing technology of electrical connectors. Electrical contacts which open and close the current flow in one or several circuits are an integral part of them. The progress in construction of electrical apparatus depends greatly on the type of electrical contacts used and their physical-mechanical properties. This fact causes the search for new solutions both in new technologies and new types of contact materials which should have arc erosion resistance, low contact resistance, high tacking resistance, high electrical conductivity, and good mechanical properties [1].

Analyzing the literature, many scientific works related to the addition of elements or compounds to the currently used contact materials (e.g., Ag–Ni, Ag–ZnO, Ag–SnO_2_) were found [2,3,4,5,6]. Due to its efficiency, price and versatility, the most commonly used material is Ag(SnO2)10 composite. A large number of scientific works related to the improvement of this composite by modifying the production technology or adding additives in the form of metals or metal oxides, which have a beneficial effect on the improvement of functional properties, have been noticed in literature [6,7,8,9,10,11]. An interesting example from these works is the use of a hybrid consisting of two contact materials working in one contact system, namely AgCe0.5 cathodes and Ag(SnO_2_)12 anodes [11]. Juszczyk et al. [5] suggest the use of a small amount (at the level of 0.25 or 0.35% by mass) of Ag_2_WO_4_ or Ag_2_MoO_4_ in order to increase the conductivity of the Ag–ZnO composite. In recent years, attempts have also been made to add CNT’s nanotubes to silver [12,13,14,15,16,17].

The authors of Reference [18] suggest the use of a silver-based contact material with the addition of TiB_2_, the particles of which were surrounded by copper oxide. In the course of the trials, it was found that CuO nanoparticles improve the electrical conductivity and arc erosion resistance of the Ag–TiB_2_ material.

The authors of Reference [19] noted the beneficial effect of rhenium addition on the reduction of arc erosion for low currents in the AgW50 material. The next paper, Reference [20], presents tests of resistance to electric arc in WCu50 high-current contacts with 2% and 5% by mass addition of rhenium. A 5-fold increase in spark erosion properties was found for the WCu50Re2 material and a 2.6-fold increase for WCu50Re5 compared to the base material WCu50. Subsequent literature items [21], however, indicate a decrease in the spark erosion properties of the AgFe9 contact material after adding a small amount of rhenium (0.5% by mass—AgFe8.5Re0.5 material). This may suggest the existence of a Re content limit in the material at which it positively affects its properties. However, the influence of differences resulting from the use of a different composite material for testing, as well as the conditions (parameters) of testing, should be taken into account. Due to the innovative nature of the work related to obtaining a new Ag–Re contact material and the lack of scientific literature related to this, the authors decided to compare the electric arc resistance of the material with the currently available and commonly used contact materials.

The use of rhenium in electrical contacts is due to its chemical properties. Metallic rhenium, despite its high melting point (3182 °C) and boiling point (5597 °C) in the presence of oxygen, at a temperature of ≥150 °C is oxidized to the Re_2_O_7_ compound [22,23]. According to Reference [24], the mentioned rhenium oxide melts in the air at 220 °C and boils at 450 °C. The molten oxide increases the contact area, reduces the contact resistance, and has a positive effect on the resistance to arc erosion and tacking of contacts.

When analyzing the usefulness of rhenium in contact materials, it was considered justified to perform tests on a silver-based material with the addition of rhenium and to analyze the possibility of replacing it with commonly used contact materials, such as: AgNi10 and Ag(SnO_2_)10. The first tests were performed with the classical powder metallurgy (mixing, pressing, sintering + extrusion, drawing, and die forged) adding 5% (AgRe5) and 10% (AgRe10) by weight of rhenium to silver [1,25,26,27,28]. In the next stage of the research, AgRe10 material was made using the Mechanical Alloying process. The reason for using this method were literature reports [29,30] indicating an increase in mechanical and electrical properties of contact materials after its application. The performed tests of spark erosion confirmed better resistance of the AgRe10 material produced with the use of MA (mixing mechanical alloying, pressing, sintering + extrusion, drawing, and die forged) as compared to the composite produced by classical powder metallurgy. Taking into account the research carried out by the authors of References [19,20,21] and the favorable results in the field of electric arc resistance obtained using the MA technology, a silver-based composite with 1% by mass addition of rhenium (according to the scheme: mixing, mechanical alloying, pressing, sintering, extrusion, drawing, die forging). The obtained results of arc resistance and the simple production technology indicate the suitability of this material (AgRe1) as a potential contact material.

## 2. Materials and Methods

**Figure 1:**
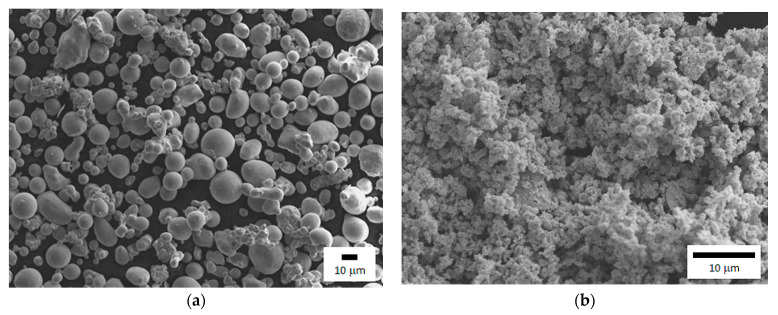
Powder morphology: (**a**) silver; (**b**) rhenium.

Metallic silver and rhenium powders were used for manufacturing composite contact materials. The silver powder was obtained by spraying liquid metal with water, while rhenium in the process of reducing ammonium perrhenate with hydrogen. Their specific surface area, grain size and morphology were tested. The specific surface area BET (Brunauer, Emmett, and Teller) multipoint was measured by means of Gemini 2360 Micromeritics, analyzer (Norcross, GA, USA). The measurement of grain size was done wet (water was used for dispersion) on Fritsch NanoTec 22” Analysette 22” (FRITSCH, Weimar, Germany) measuring unit according to Fraunhofer theory, with a measuring range of 0.1 µm to 501.48 µm. Particle morphology was observed on an x-ray microanalyzer JEOL 8230 (Akishima, City, Japan). The test results are shown in Table 1. The BET multipoint specific surface area of silver powder is around 3 times smaller than of the rhenium powder. This can be connected with the size of particles, as well as the method of obtaining them, which affects development, shape, and size of the grains. The average size of silver particles is 12 times bigger than of rhenium, which was confirmed by morphology tests. Silver powder grains are both spherical and globular in shape, with a significant advantage of the second option (Figure 1a). Rhenium powder particles are fine and irregular (Figure 1b).

Mixtures were prepared using the powders. Their chemical composition is presented in Table 2.

**Figure 2:**
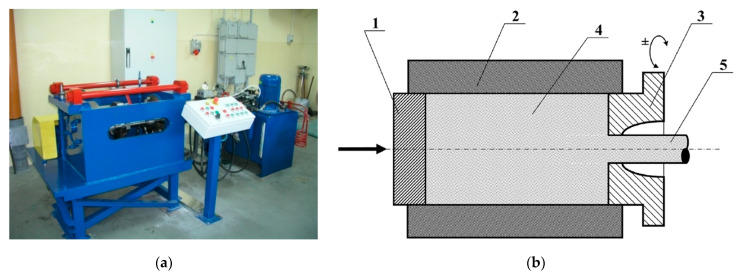
The Kobo hydraulic extrusion press for extruding metals and alloys: (**a**) general view; (**b**) principle of operation 1 = punch, 2 = container, 3 = a die rotating on both sides, 4 = starting material, 5 = final product (**a**) is reprinted with permission from ref. [28]. Copyright 2016 Polish Academy of Sciences. (**b**) is reprinted with permission from ref. [31]. Copyright 2013 Łukasiewicz Research Network–Metal Forming Institute.)

The powder mixtures, depending on the production method, were pressed and sintered or subjected to mechanical alloying, pressing, and sintering. The mechanical alloying process was used in case of the material with a 1% and 10% Re by mass. The other composites (also part of the material with 10% Re by mass) were manufactured by means of classical powder metallurgy (mixing, pressing, sintering + extrusion, drawing, die forged). The mechanical alloying process was carried out in a ball mill under protective argon atmosphere. All mixtures, apart from the AgRe1, were cold isostatically pressed into rollers with a diameter of around 18 mm (AgRe5, AgRe10) and 20 mm (the AgRe1 material). Next, they were sintered and extruded on a Kobo hydraulic press [28,31], as shown in Figure 2. In the case of the AgRe5 and AgRe10 materials, obtained by means of classical powder metallurgy the extrusion ratio λ was 20, the wire was extruded to a 4 mm in diameter. After the extrusion process, the material was consolidated by drawing and heat treatment to the size enabling manufacturing of electrical rivets. The procedures were similar for powders subjected to mechanical alloying, with the difference that the extrusion ratio λ was 52 and the blank wire was extruded to 2.5 mm diameter (in the case of AgRe10 composite; this procedure is marked as method 1). In addition, a part of the AgRe10 material subjected to consolidation in the isostatic press and the free sintering process in the continuous furnace was additionally sintered under pressure using the SPS-Spark Plasma Sintering method (750 °C, 10 min, 35 MPa) and marked further in the article as method 2. After plastic consolidation, the wire was die forged into electrical contacts.

**Figure 3:**
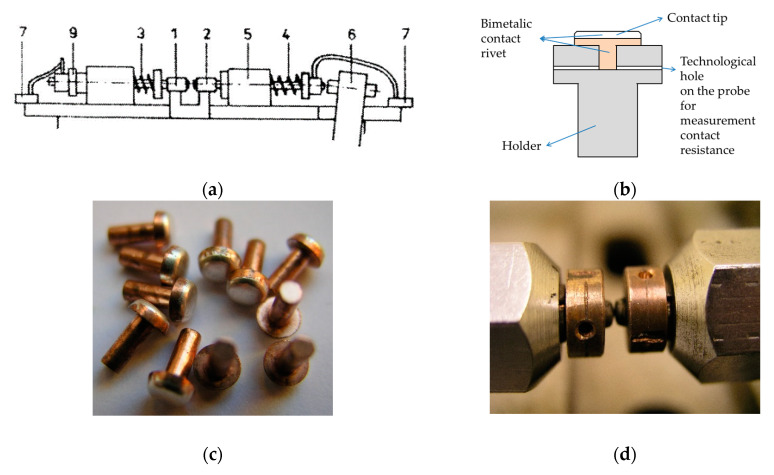
Construction of a single current circuit of the testing device. (**a**) scheme: 1 = fixed contact, 2 = mobile contact, 3 = contact spring, 4 = returnable spring, 5 = mobile contact guiding, 6 = live, 7 = current terminals, 9 = nut adjusting the clamping force, (**b**) the tested contact mounted in a copper holder, (**c**) contact rivets, (**d**) view of the contact of electric rivets (**c**) is reprinted with permission from ref. [27]. Copyright 2016 Polish Academy of Sciences. (**a**,**d**) are reprinted with permission from ref. [28]. Copyright 2016 Polish Academy of Sciences.)

Spark erosion tests and contact resistance measurement were conducted on model equipment for examination of arc erosion. The device has six separate current circuits, presented in Figure 3a. The measurement consists of connecting and disconnecting current circuits through a set of tested contacts mounted in appropriate holders. The test was performed with different voltage characteristics (DC and AC). The current value was 10 A for DC and 60 A for AC. The test parameters are shown in Table 3. Resistance measurements are carried out in the system without applied voltage of 230 V. Bimetallic contact rivets with convex heads (Figure 3b–d) were used for testing (10BW4/1.5: 10—radius of the contact head, B—bimetallic, W—convex, 4—diameter of the contact head, 1.5—contact head height). The contact layer thickness is about half of the height of the rivet head (approximately 0.6–0.7 mm). The initial connection of the base material (Cu) and the contact material takes place by friction-impact forging. After the electric rivet is formed, the materials are diffused during their heat treatment.

The AgNi10 and Ag(SnO_2_)10 composite materials, available for retail sale, were used as a reference material. The number of switching cycles was 50,000 for DC and 15,000 for AC. The differences in their numbers resulted from the fact that the contacts were damaged when a larger current (60 A) was used at 15,000 switching cycles. Electric arc resistance was defined as the contact mass loss after a strictly defined number of switching cycles. For this purpose, the contacts were removed from the device and weighed, and then the mass loss was determined.

## 3. Results

**Figure 4:**
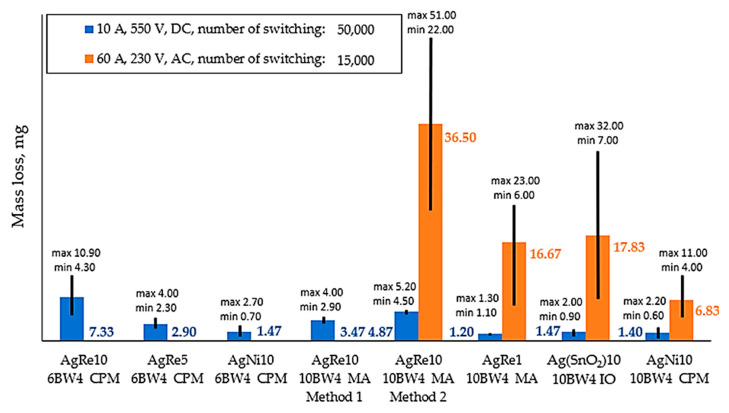
A graphic representation of erosion measured for the contacts under test (average measurements values), CPM = classical powder metallurgy, MA = mechanical alloying, IO = internal oxidation.

**Figure 5:**
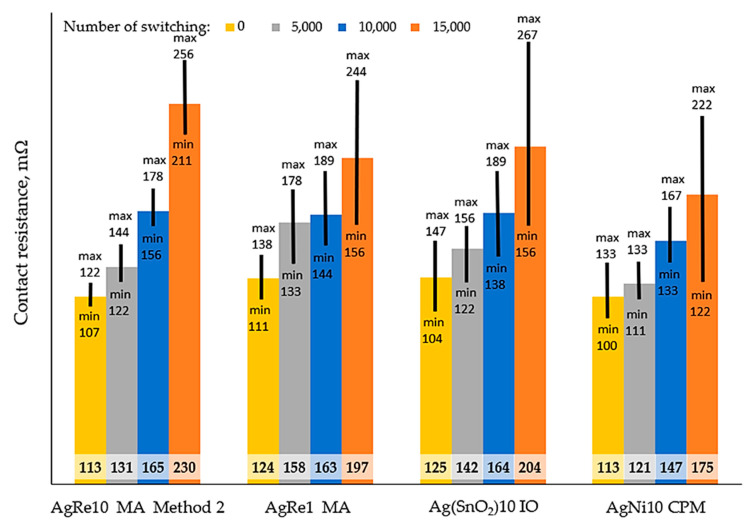
The results of the contact resistance tests, MA (mechanical alloying), IO (internal oxidation), CPM (classical powder metallurgy).

The results of the electric arc resistance tests are presented in Figure 4. The contact resistance test was carried out only for rivets for which the electric arc resistance was tested in an alternating current system with a value of 60 A. The test results for 0, 5000, 10,000, and 15,000 switching cycles are shown in Figure 5.

**Figure 6:**
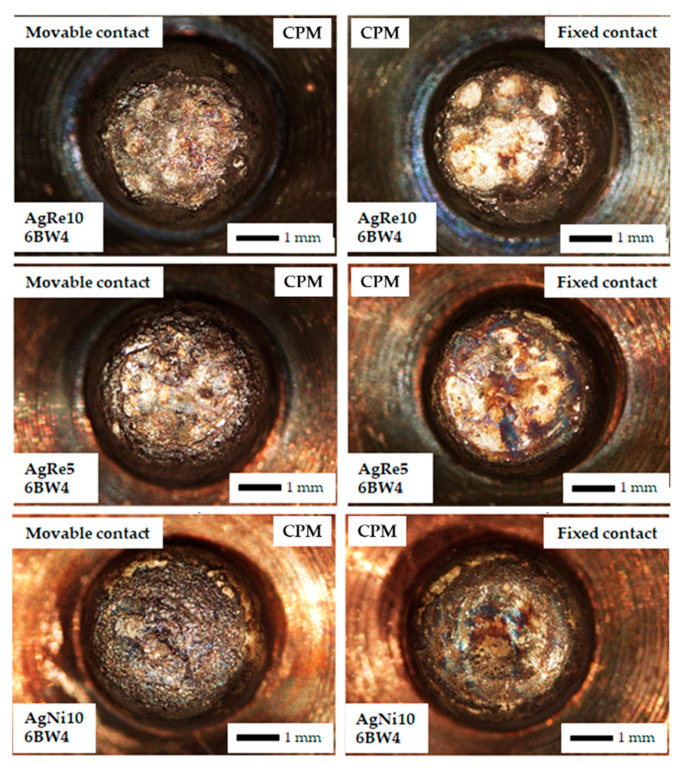
Macro photographs of contact surface after the arc erosion testing DC current = 10 A, voltage = 550 V, CPM = classical powder metallurgy.

**Figure 7:**
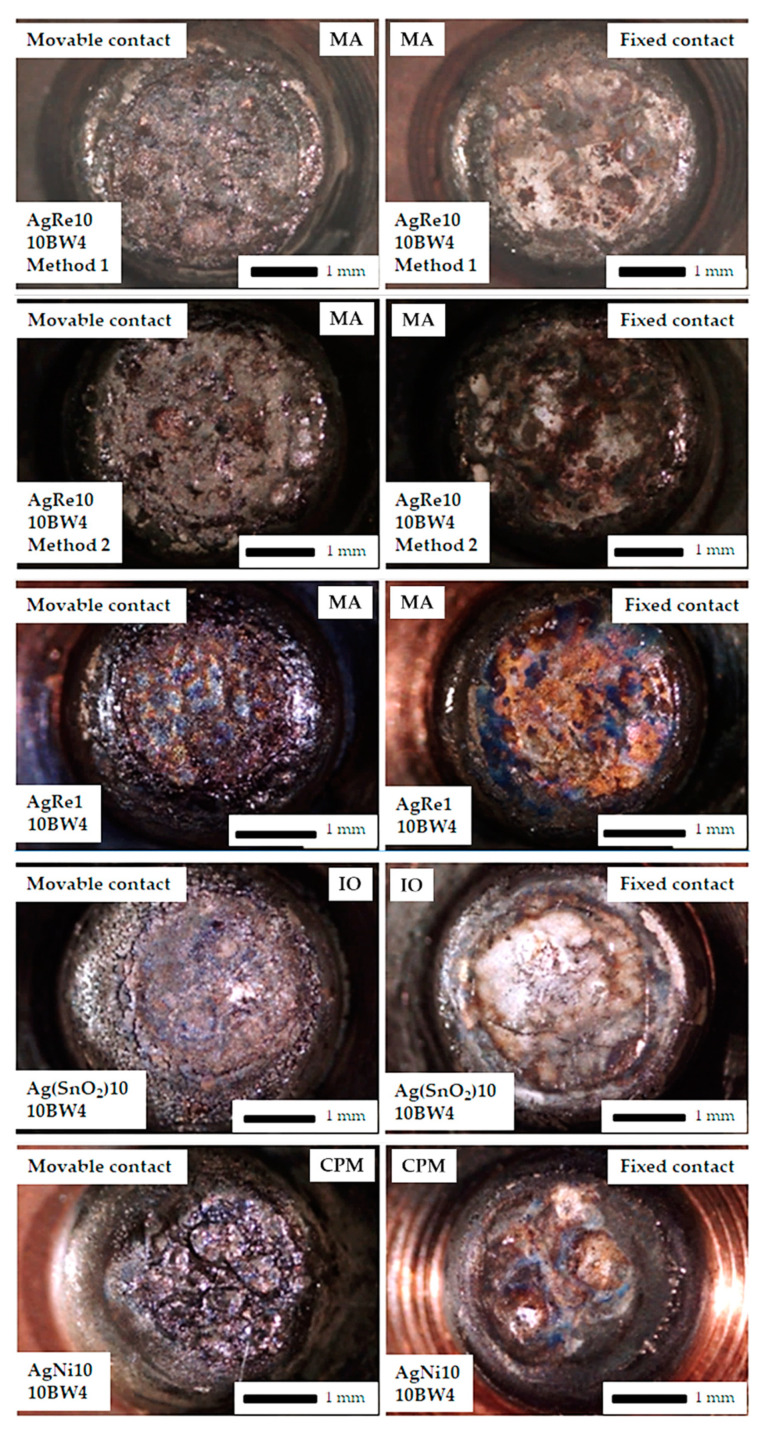
Macro photographs of contact surface after the arc erosion testing DC current = 10 A, voltage = 550 V, MA = mechanical alloying, IO = internal oxidation, CPM = classical powder metallurgy.

**Figure 8:**
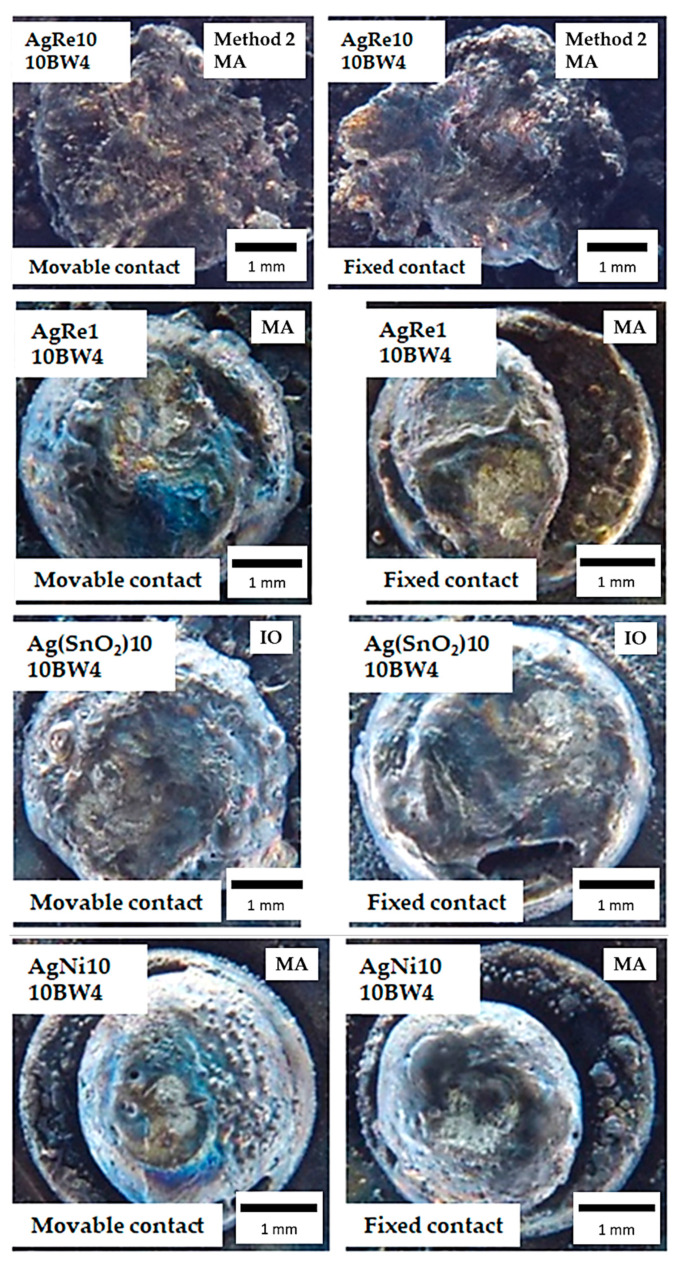
Macro photographs of contact surface after the arc erosion testing AC current = 60 A, voltage = 230 V, MA = Mechanical Alloying, IO = Internal Oxidation.

Figure 6, Figure 7 and Figure 8 show a view of the contact face surfaces after the experiment. For the 10 A DC, the changes are not significant and the surfaces do not have significant craters related to the contact material melting and evaporation. The greatest damage was observed in the case of AgNi10 material (CPM—Classical Powder Metallurgy, 10BW4/1.5 and 6BW4/1.5). In the case of Ag(SnO_2_)10 material, we notice slight cracks in the material on the contact surface (IO-Internal Oxidation, 10BW4/1.5). Contacts containing the addition of rhenium have a surface free from cracks and degradation due to melting. All the mentioned changes do not substantially affect the further function of the contacts. Another situation occurs after applying 60 A AC. The contacts have been completely destroyed. The greatest disintegration of the working surface of the contacts was noted for the AgRe10 material. Erosion products related to the action of electric arc have been found on the contact surface of each type of contact material. In the case of AgRe1 and AgNi10 materials, physical separation of the contact material from the base material (copper) was observed.

## 4. Discussion

The results obtained in terms of resistance to electric arc for the system load with an alternating current of 60 A indicate that the AgNi10 composite (mass loss 6.83 mg) has the best spark erosion properties, followed by AgRe1 (mass loss 16.67 mg), and then followed by Ag(SnO_2_)10 (mass loss 17.83 mg). For the DC system, the AgRe1 composite (1.20 mg mass loss) had the best resistance to electric arc, whose properties, in this respect, are comparable with reference materials, such as: AgNi10 (mass loss 1.40 mg) and Ag(SnO_2_)10 (mass loss 1.47 mg). For composites produced by classical powder metallurgy (CPM), AgNi10 (mass loss at the level of 1.47 mg) had the best resistance to electric arc.

In order to compare the spark erosion properties of materials better, the ratio of their mass loss defined as the ratio of the selected material to the base material was calculated (AgNi10). This ratio was introduced due to the differences resulting from different diameters of working surfaces of electrical contacts (for materials manufactured by means of CPM (Classical Powder Metallurgy) the diameter is 6, whereas for others—10). Table 4 shows its value, a value under 1 indicates better spark erosion properties of the composite material than of the base material (AgNi10), if it is higher the situation is the opposite. The analysis of the test results shows that AgRe1 has the best spark erosion properties when connected to direct current load, whereas the AgRe10 material manufactured by means of classical powder metallurgy has the worst test result. It was observed that, after applying mechanical alloying, its electrical arc resistance increased two-fold.

It is interesting that the electric arc resistance decreased with the increase in the amount of rhenium in the material. Presumably, it results from the chemical properties of Re which oxidizes to the Re_2_O_7_ compound as the temperature increases.

When testing the contact resistance, its increase was observed along with the number of switching times for all tested contact materials. Presumably, it is related to the degradation of the working surfaces of the contacts during arcing, the evaporation of materials or the formation of oxides. For AgRe1 and Ag(SnO_2_)10 material, this increase is at a similar level. The contact material AgNi10 had the lowest contact resistance among the tested composites; the next one was AgRe1, then Ag(SnO_2_)10, while the highest, thus being the lowest conductivity, had the AgRe10 material made by method 2.

The phenomenon of surface evaporation (heating the surface to the evaporation temperature of the material) has the greatest impact on arc erosion at low currents, while, in the case of using high currents, the determining phenomenon causing the degradation of contact materials is the ejection of molten droplets due to the presence of plasma beams (pressure generated by the electric arc) [32]. Such a large degradation of the AgRe10 con-tact material in the case of using the AC current of 60 A may additionally be associated with the ejection of molten metal oxide (Re_2_O_7_), which, in turn, causes a large degradation of the contact surfaces and a change in the shape of the contact material. In the case of small currents (Figure 6 and Figure 7), we observe small craters on the contact surfaces due to numerous melts. They are small and do not substantially affect the functioning of the contacts. The aforementioned material losses are related to the heating of the contact materials during the switching on and off of the contact, as well as the operation of the contact itself (current flow). The aforementioned process is influenced, among others, by Physical phenomena that occur during the operation of the contact, such as: Joule heating, the occurrence of an electric arc when switching on and off the contact, heating the contact material with plasma beams, and the occurrence of chemical reactions (endothermic and exothermic).

Figure 4 and Figure 5 show the minimum and maximum values, and Table 5 and Table 6 show the standard deviation, the coefficient of variation (CV) of the measurement. In the case of electric arc erosion resistance for direct current, the AgRe10 material obtained using the mechanical alloying process and the Spark Plasma Sintering process is characterized by the lowest value of the CV coefficient. The second in line is AgRe1 material, whose value of the coefficient of variation is 8.33%. As can be seen, the CV when rhenium is used is substantially lower than the other materials tested (AgNi10 and Ag(SnO_2_)10). The material produced by classical powder metallurgy for which the CV value = 45.47% is an exception. Analyzing the influence of mechanical alloying on the repeatability of the results of spark erosion tests, a positive influence of the application of this process was noticed. This may be related to the positive influence of mechanical alloying, which influences rhenium dispersion in the composite, grain refinement, and the formation of a nanocrystalline structure in silver. The chemical properties of Re may additionally influence such good reproducibility of results in the case of composites with rhenium addition. As previously mentioned, rhenium is oxidized at low-temperature to the compound Re_2_O_7_, which melts at low temperature, causing an increase in the contact surface. This can be seen in particular during resistance measurements. During the tests, good reproducibility of results was observed for composites with the addition of rhenium (excluding the AgRe1 composite case for 15,000 switching cycles), especially for the AgRe10 material.

The results of the electric arc resistance tests and contact resistance for low rhenium contents indicate the possible usefulness of the new composite material in the design of all kinds of electrical and electronic devices. Some issues with its application may arise from the retail price of rhenium; however, for special applications where product economy is of secondary importance, it can be a good alternative to any kind of other contact material.

The presented Ag–Re material may be useful for applications in new electrical devices, the nature of which changes from resistive to capacitive.

The uncomplicated production technology of Ag–Re composite contact material, especially in relation to the production technology of Ag(SnO_2_) material obtained by internal oxidation (IO), presents it as a competitive contact material.

## 5. Conclusions

As part of the research, the following conclusions were formulated:

1. The addition of rhenium in the amount of 1% by mass improves the Ag–Re composite resistance to electric arc.
2. The introduction of a larger amount of rhenium to the Ag–Re composite (10% by mass) reduces the electric arc resistance.
3. The use of the mechanical alloying process in the Ag–Re composite production process increases its resistance to electric arc.
4. The obtained results in the field of electric arc resistance prove that AgRe1 composite is more resistant to electric arc than the commonly used contact materials Ag(SnO_2_)10 and AgNi10 (for DC current, 10 A) and the Ag(SnO_2_)10 composite (for AC 60 A).

## Figures and Tables

**Table 1:** Properties of powders used for manufacturing contact materials.

Metal	Specific Surface Area BET Multipoint, m^2^/g	Average Powder Size, µm
Ag	0.056 ± 0.001	24.33 ± 0.49
Re	1.448 ± 0.016	1.69 ± 0.04

**Table 2:** Chemical composition of the mixtures.

Contact Material	Ag, % by Mass	Re, % by Mass
AgRe1	99	1
AgRe5	95	5
AgRe10	90	10

**Table 3:** Electric and mechanical parameters of the arc erosion resistance tests.

Parameter	Direct Current, DC	Alternating Current, AC
Current, A	10	60
Voltage, V	550	230
Distance between contacts, mm	6	5
Force of pressure, N	10	10
Number of switching cycles	50,000	15,000

**Table 4:** Mass loss ratio.

Material (A)	Reference Material (B)	Rivet Shape	Technology Type	Value and Type of Load	Mas Loss A, mg	Mas Loss B, mg	Mass Loss Ratio, (mas loss A)/ (mas loss B)
AgRe10	AgNi10	6BW4/1.5	CPM	10 A, DC	7.33	1.47	5.0
AgRe5					2.90		2.0
AgRe10–method 1		10BW4/1.5	MA		3.47	1.40	2.5
AgRe10–method 2			MA + SPS		4.87		3.5
AgRe1			MA		1.20		0.9
AgRe10–method 2				60 A, AC	36.50	6.83	5.3
AgRe1					16.67		2.4
CPM = classical powder metallurgy; MA = mechanical alloying; SPS = spark plasma sintering							

**Table 5:** Average and standard deviation and coefficient of variation of mass loss, number of switching: 50,000 for DC and 15,000 for AC.

Material	Rivet Shape	Technology Type	Value and Type of Load	Mass Loss, mg		Coefficient of Variation, %
				Average	Standard Deviation	
AgRe10	6BW4/1.5	CPM	10 A, DC	7.33	3.33	45.47
AgRe5				2.90	0.95	32.89
AgNi10				1.47	1.08	73.23
AgRe10–method 1	10BW4/1.5	MA		3.47	0.55	15.88
AgRe10–method 2		MA + SPS		4.87	0.35	7.22
AgRe1		MA		1.20	0.10	8.33
Ag(SnO_2_)10		IO		1.47	0.55	37.49
AgNi10		CPM		1.40	0.80	57.14
AgRe10–method 2		MA + SPS	60 A, AC	36.50	14.50	39.73
AgRe1		MA		16.67	9.29	55.75
Ag(SnO_2_)10		IO		17.83	12.83	71.96
AgNi10		CPM		6.83	3.69	53.99
CPM = classical powder metallurgy; MA = mechanical alloying; SPS = spark plasma sintering						

**Table 6:** Average and standard deviation and coefficient of variation of contact resistance, AC, 60 A.

Number of Switching	Contact Resistance, mΩ		Coefficient of Variation, %
	Average	Standard Deviation	
**AgRe10–method 2, 10BW4/1.5, MA + SPS**			
0	113.00	7.94	7.02
5000	131.00	11.53	8.80
10,000	165.00	11.53	6.99
15,000	230.00	23.30	10.13
**AgRe1, 10BW4/1.5, MA**			
0	124.00	13.53	10.91
5000	158.00	22.91	14.50
10,000	163.00	23.30	14.30
15,000	197.00	44.31	22.49
**Ag(SnO_2_)10, 10BW4/1.5, IO**			
0	125.00	21.52	17.21
5000	142.00	17.78	12.52
10,000	164.00	25.51	15.56
15,000	204.00	57.00	27.94
**AgNi10, 10BW4/1.5, CPM**			
0	113.00	17.58	15.56
5000	121.00	11.14	9.20
10,000	147.00	17.78	12.09
15,000	175.00	50.27	28.73
CPM = classical powder metallurgy; MA = mechanical alloying; SPS = spark plasma sintering			

## Data Availability

Data sharing is not applicable to this article.
